# Studies on the functionality of the TC-NER ERCC6-M1097V protein variant frequently found in Louisiana patients with PCa upon UV damage

**DOI:** 10.3389/fonc.2025.1679379

**Published:** 2026-02-03

**Authors:** Oluwatobi Ogundepo, Arrigo De Benedetti

**Affiliations:** Department of Biochemistry and Molecular Biology, Louisiana State University Health Shreveport, Shreveport, LA, United States

**Keywords:** androgen deprivation therapy (ADT), CDDP (cisplatin), cyclobutane pyrimidine dimers (CPDs), homologous recombination repair (HRR), inter/intra strand crosslinks (CDDP-ISLs), metastatic castration-resistant prostate cancer (mCRPC), mismatch repair (MMR), prostate cancer (PCa)

## Abstract

ERCC6, also known as CSB (Cockayne Syndrome B), is a key protein involved in transcription-coupled nucleotide excision repair (TC-NER), a DNA repair process that removes lesions blocking RNA polymerase. ERCC6’s multifaceted roles include chromatin remodeling, transcription regulation, oxidative stress response, and coordination with other DNA repair proteins. Mutations in ERCC6 lead to Cockayne Syndrome and other neurodegenerative disorders, but some variants, such as M1097V, have been associated with cancer risk, particularly prostate cancer (PCa) in African Americans (AAs) in Louisiana. Recent studies have explored the functional impact of ERCC6 variants in PCa, especially among AAs, who face higher incidence and more aggressive forms of the disease. A notable finding is that the M1097V variant increases cellular tolerance to UV damage, suggesting not only a possible evolutionary benefit but also a potential risk for mutagenesis when exposed to complex environmental carcinogens. Other ERCC6 mutations, such as S636N (also only found in Louisiana PCa), located near regulatory regions, may alter repair activity, though their effects remain unclear. Given the high mutation burden in mismatch repair (MMR) and NER genes observed in AA patients with PCa, a synthetic lethality strategy targeting both TC-NER and homologous recombination repair (HRR) pathways could be effective. This includes combining agents like CDDP (cisplatin) with inhibitors of RAD54, such as J54. These approaches may offer alternatives to androgen deprivation therapy (ADT), which is often ineffective in advanced or treatment-resistant PCa common among AA men. This work underscores the importance of integrating genetic, environmental, and therapeutic insights to address PCa disparities.

## Introduction

Prostate cancer (PCa) is the most commonly diagnosed non-cutaneous malignancy and the second-leading cause of cancer-related death among men in the United States. Notably, African American (AA) men bear a disproportionate burden of this disease compared to men of European ancestry [Caucasians (CC)], exhibiting higher incidence rates, earlier onset, and increased mortality from disease that is refractory to treatment (3). This is, to some extent, explained by genetic differences in some cases attributed to alterations of the “repairome” (4). While socioeconomic, environmental, and healthcare access disparities contribute to these differences, accumulating evidence suggests that biological factors, including genomic and molecular alterations, may play a critical role in driving the aggressive phenotype observed in AA patients.

The DNA damage response and repair pathways (DDRR), collectively known as the “repairome,” are essential for maintaining genomic stability and preventing malignant transformation. Defects in DNA repair mechanisms are a well-established hallmark of cancer, contributing to increased mutational burden, chromosomal instability, and therapeutic resistance. In PCa, alterations in key DNA repair genes—such as *BRCA1*, *BRCA2*, *ATM*, and *MLH1*—have been associated with tumor progression, poor prognosis, and sensitivity to targeted therapies, including PARP inhibitors and platinum-based agents. Inflicting DNA damage and enhancing apoptosis of cancer cells are the mechanistic strategies of all radiotherapeutic (e.g., external beam radiation therapy, brachytherapy, and radium-223) and some chemotherapeutic [PARP inhibitors (PARPis), topoisomerase inhibitors, platinum-based therapy (5), and DNA crosslinking agents] methods (6, 7). The relative success of PARPi for metastatic castration-resistant prostate cancer [mCRPC (PROfound (8) and TRITON2 (9))] is defining new biological and more effective treatment options for previously unmanageable PCa, and while cisplatin (CDDP)-based therapy is currently not the treatment of choice for PCa, partly due to its significant renal toxicity [which can be faithfully recapitulated in mice (10)], the current trend suggests that lower dosing in combination with inhibitors of DNA damage repair could be quite effective (5). Helicases are central to maintaining genomic integrity, mediating processes such as DNA replication, repair, transcription, and chromatin remodeling. Dysregulation of helicase activity has been implicated in various cancers, yet their specific contributions to PCa remain underexplored. Preliminary studies suggest that certain helicases are overexpressed or mutated in PCa and may influence key oncogenic pathways, including androgen receptor (AR) signaling, genomic instability, and cellular stress responses. Furthermore, therapies targeting key aspects of DNA repair mechanisms, including NER, BER, and HRR with PARPis, particularly for PCa cases manifesting BRCAness, have proven to be the greatest clinical advancement since the use of Androgen Receptor Signaling Inhibitors (ARSI). ERCC6 is a helicase whose primary function is mediating the dislodging of transcription Elongation Complexes (EC) stuck at DNA-distorting lesions. Mutations in this protein reduce the rate of rRNA synthesis, and cancer cells with a functional knockout display detrimental growth effects. This protein also acts as an anti-apoptotic factor, tipping the cell towards proliferation and survival, while loss of function results in cell cycle arrest and senescence via its interaction with p53, and in the rare Cockayne syndrome, in progeria.

Emerging studies have identified race-specific differences in the frequency, type, and functional consequences of repairome alterations in prostate tumors. AA men may harbor unique germline and somatic variants in DDR genes, as well as distinct patterns of gene expression and epigenetic regulation within DNA repair networks. Among these molecular differences that may underlie the observed disparities in tumor biology and response to therapy, yet remain incompletely characterized due to the historical underrepresentation of AA men in genomic studies, is a specific polymorphism in ERCC6 (M1097V) (11), although this haplotype was reported for a greater prevalence of this mutation in AA-PCa (21% frequency *vs*. 1% for CC) (11). This was determined from only a small number of patients (11). However, this same polymorphism was identified as a significant risk for ontogeny and worse outcomes in meta-analyses of other types of cancer worldwide (12–14).

In this study, we introduced this genomic mutation via CRISPR/Cas9 in a panel of common PCa cell lines, including PCa2 cells derived from an AA patient, and we investigate the consequences for the repair of lesions requiring transcription-coupled nucleotide excision repair (TC-NER) (UV and cisplatin resistance) as a first assessment of the possible altered interaction of this mutant protein with the environment that these individuals may be exposed to. For instance, in Louisiana, PCa disparity is far more prevalent, with higher incidence and worse overall survival (OS) that can be attributed to both genetic components and dietary habits (15), compounded by much greater health risks from a regional toxic environment that was built to disproportionally impact the AA population (16).

## Materials and methods

### Site-directed mutagenesis: Crispr SDM

The guide RNA and donor DNA were designed and purchased via the Thermofisher Invitrogen TrueDesign Genome Editor (https://www.thermofisher.com/us/en/home/life-science/genome-editing/invitrogen-truedesign-genome-editor.html). Following the protocol, the cells were seeded to 70% confluency. After 24 h, in tube 1, TrueCut Cas9 protein, TrueGuide sgRNA, and donor DNA (**Table 1**) were diluted with Cas9 Plus Reagent in Opti-MEM solutions, and in another tube, CRISPRMAX reagent was diluted in Opti-MEM medium. The reagents were mixed, incubated for 10 min, and added to the cells. After 48 h, single-cell clones were created and screened.

**Table 1:** Primers list.

Guide RNA	GTTACATTACTACTCATGTG
Donor DNA or TrueTag Sequence	CTAATCGAAGTGATCCTTTGAAAGATGACCCTCACGTGAGTAGTAATGTAACTAGCAATGATAGGCTTGGAGA
ERCC6_SEQ_FWD_P1	GTTCAGACACCCAAATGCCA
ERCC6_SEQ_FWD_P2	AAACGCAAGAAGTTCCCTGC
ERCC6_SEQ_REV_P1	AGGGTCTCTTCTTCTGCCAC
ERCC6_SEQ_REV_P2	CTTCTGTTTGAGCCTGGCTG
Target Sequence	GTTACATTACTACTCATGTG
PAM	AGG
Score	97.09
Genomic Location	chr10[49470654]

### Plasmid vector SDM

The same M1097V mutation was introduced in expression vector RC219020 (OriGene: https://www.origene.com/catalog/cdna-clones/expression-plasmids/rc219020-csb-ercc6-nm-000124-human-tagged-orf-clone) using the QuickChangeII SDM kit (Agilent cat#200523). Transient transfections were carried out with Lipefectamine-3000 according to the manufacturer for 48 h without medium replacement.

SDM FW primer: CCTTTGAAAGATGACCCTCGCATGAGTAGTAATGTAACTAGCAATGA

### Gel electrophoresis

Agarose gels (1%) were prepared with 1× TAE buffer (from a 50× stock solution; EDTA disodium salt 372.24 g/mol, Tris 121.14 g/mol, glacial/acetic acid 60.05), after which fresh EtBr was added before use. Electrophoresis was performed with a constant 80 V. After electrophoresis, the gel image was captured by the BIORAD ChemiDoc Imaging System. PCR-restriction fragment length polymorphism (RFLP) Hin1II (NlaIII) cleavage sites were identified in our expected amplicons using the New England Biolabs NEBcutter tool. Following PCR amplifications of the ERCC6 M1097V region, 20% of the reaction volume was incubated with the enzyme and run on a gel electrophoresis as described in the gel electrophoresis method, followed by confirmatory sequencing.

### Dot blot (DNA Southwestern blot)

A total of 50,000 cells were plated and incubated for 24 h and then exposed to UV for 30 s. They were then allowed to recover for different time points, after which the cells were lysed using the X-Amp DNA reagent, Cat. No. IB47441; dot blotted on Millipore INYC00010 IMMOBILON via a dot blot apparatus (Bio-Rad); and baked at 80°C for 30 min. The membrane was dipped in water and then 0.1% methylene blue and incubated with a primary antibody, mouse monoclonal anti-cyclobutane pyrimidine dimer (CPD) [C3B6]–Absolute antibody diluted 1:1000 in 0.5% BSA overnight. The membrane was washed twice gently in 0.02% TBST for 10 min and incubated in a secondary antibody, anti-mouse IgG, HRP-linked antibody (Cell Signaling Technology) 1:5,000 in 0.5% BSA in 0.02% TBST, and then washed three times and visualized using the BIORAD ChemiDoc Imaging System.

### Proliferation assay

Cells were seeded into Greiner Cell culture Microplate, 96 wells, Ps, F-Bottom 655180 at 50% confluency (10,000 cells). After 24 h, the cells were exposed to different doses of UV and placed into IncuCyte S3. The UV source is generated by a Stratalinker that was calibrated at an intensity of 200 mJ/cm^2^/10 s exposure. Cell growth was monitored using the Incucyte^®^ Live-Cell Analysis System to capture phase contrast images every 4 h and analyzed using the integrated confluence algorithm.

### ATPase assay

This was carried out with ADP-Hunter (DiscoveX) according to the manufacturer’s protocol (https://www.discoverx.com/catalog/adp-hunter-plus?item=90-0083) and using ~50 ng of IP-purified ERCC6 [Myc Tag Monoclonal Antibody (Myc.A7) (MA1-21316)] and ±50 ng of plasmid DNA. Since we wished to monitor the reactions kinetics at steady state (with and without DNA), and not the initial burst of ATP consumption for intramolecular domain reorganization and/or DNA binding, the *t*_0_ time point was selected as after 15 min of pre-incubation at 37°C, as earlier described in (17), and likewise nonspecific RLU from reagents alone was <3,000.

### Statistical analysis

GraphPad Prism 9 was used to perform statistical analysis, and Microsoft Excel software (Version 16.88) was used for data handling. Results are viewed as mean ± standard error of the mean (SEM). Statistical significance was calculated by a Student’s *t*-test when comparing the mean between two groups. *p*-values  <  0.05 were considered significant (*p*-value: *p < 0.05, **p < 0.01, and ***p < 0.001) for comparison of growth slopes recovery after linear regression curve generated by GraphPad from Incucyte raw data that included replicas, degrees of freedom, and data time point numbers to generate the slopes.

## Results

**Figure 1:**
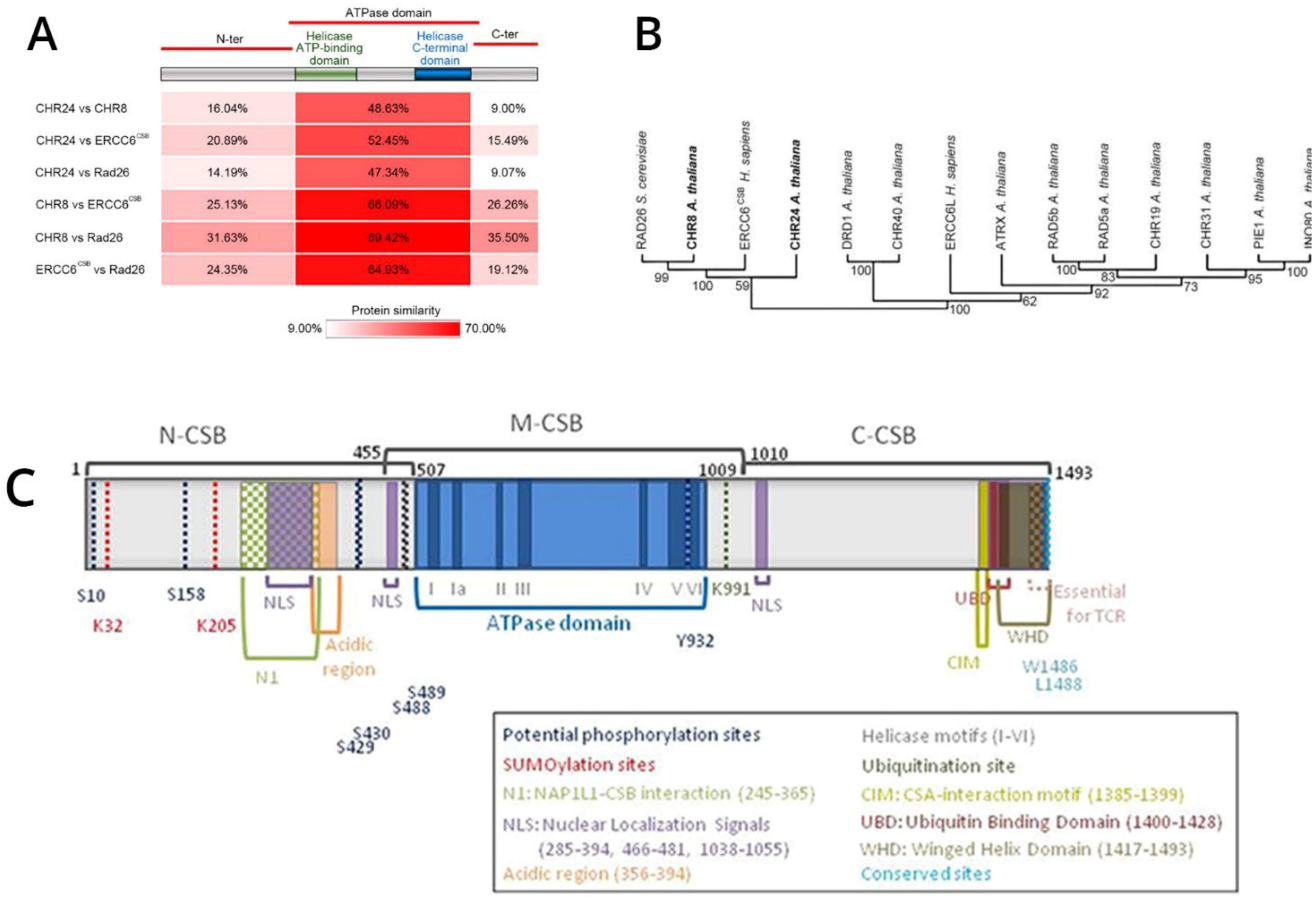
ERCC6 is a conserved protein in human, mouse, plant (e.g., *Arabidopsis thaliana*), and yeast (e.g., *Saccharomyces* spp.). **(A)** A table summarizing and comparing the conserved regions in ERCC6 and similar proteins taken from (1), **Supplementary Figure S5**. **(B)** Phylogenetic relationship between ERCC6 and related protein, including CHR8/24 in plant and RAD26 in yeast, taken from (1). ERCC6 schematic structure taken from (2). **(C)** ERRCC6 domains.

**Figure 2:**
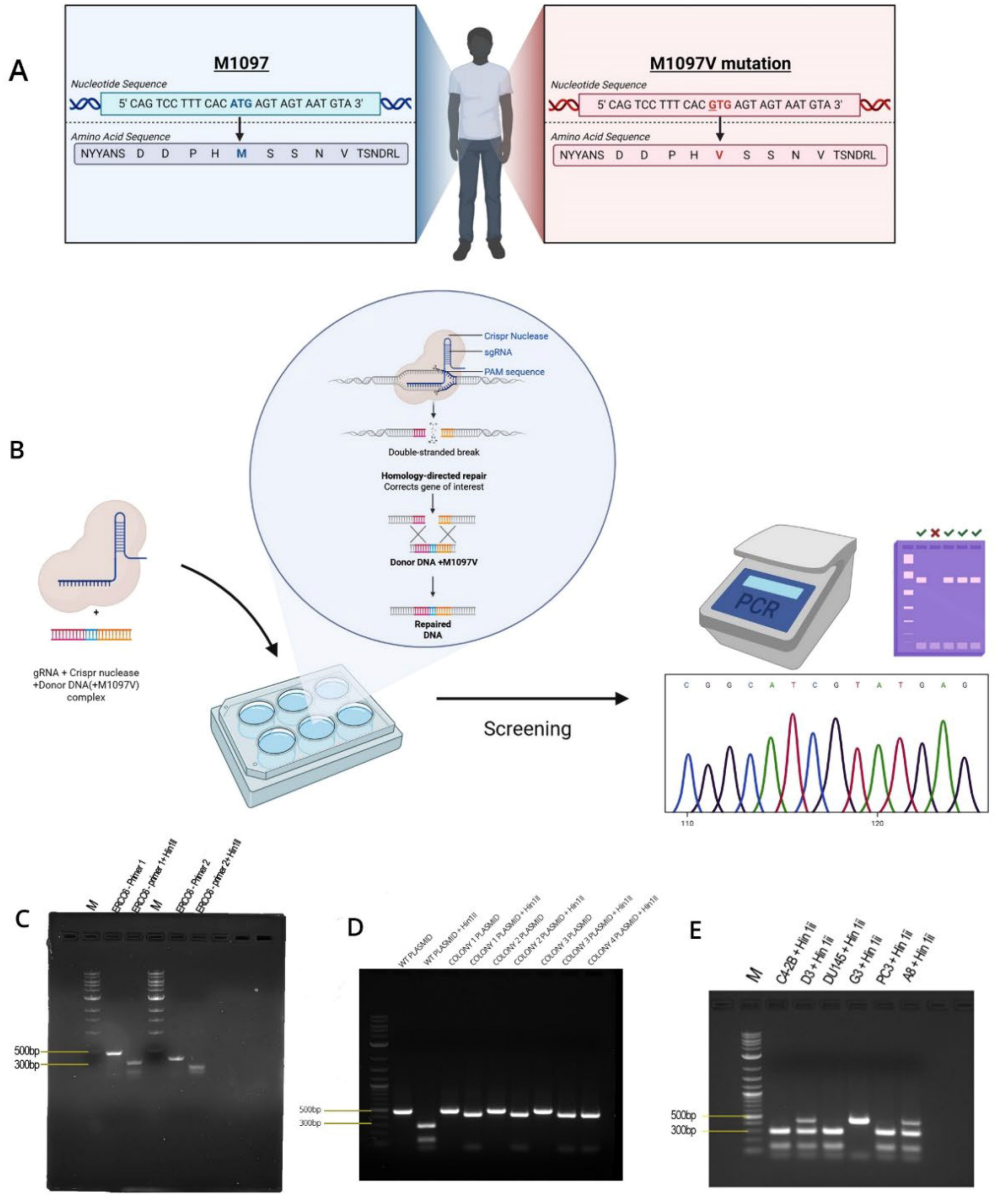
Generation and validation of the M1097V knock-in in prostate cancer cells using the CRISPR/Cas9 editing system. **(A)** A schematic diagram of the recurring ERCC6 M1097V mutation, created with BioRender. **(B)** A schematic diagram of the CRISPR/Cas9 editing system, made with BioRender. **(C)** PCR amplification of the M1097V region and cleavage with HIN1II. **(D)** Confirmation of the cleavage site using the ERCC6 plasmid from Origene. **(E)** Clone confirmation after generating the M1097V variants with CRISPR/Cas9 via PCR-RFLP. Graphical sketch prepared with BioRender.

ERCC6 is an essential, highly conserved gene from yeast to mammals (**Figure 1**). Given its essentiality, mutations are rare and almost absent from the PCa TCGA-500 database. Therefore, the unusual frequency of the M1097V variant, more so in AA, may be a peculiarity of the Louisiana population. Starting from the genomic site-directed mutagenesis (SDM) work, we have introduced the M1097V mutations in the indicated cell lines (**Figure 2**) via CRISPR-mediated recombination and obtained hetero- and homozygous (bi-allelic) SDM.

**Figure 3:**
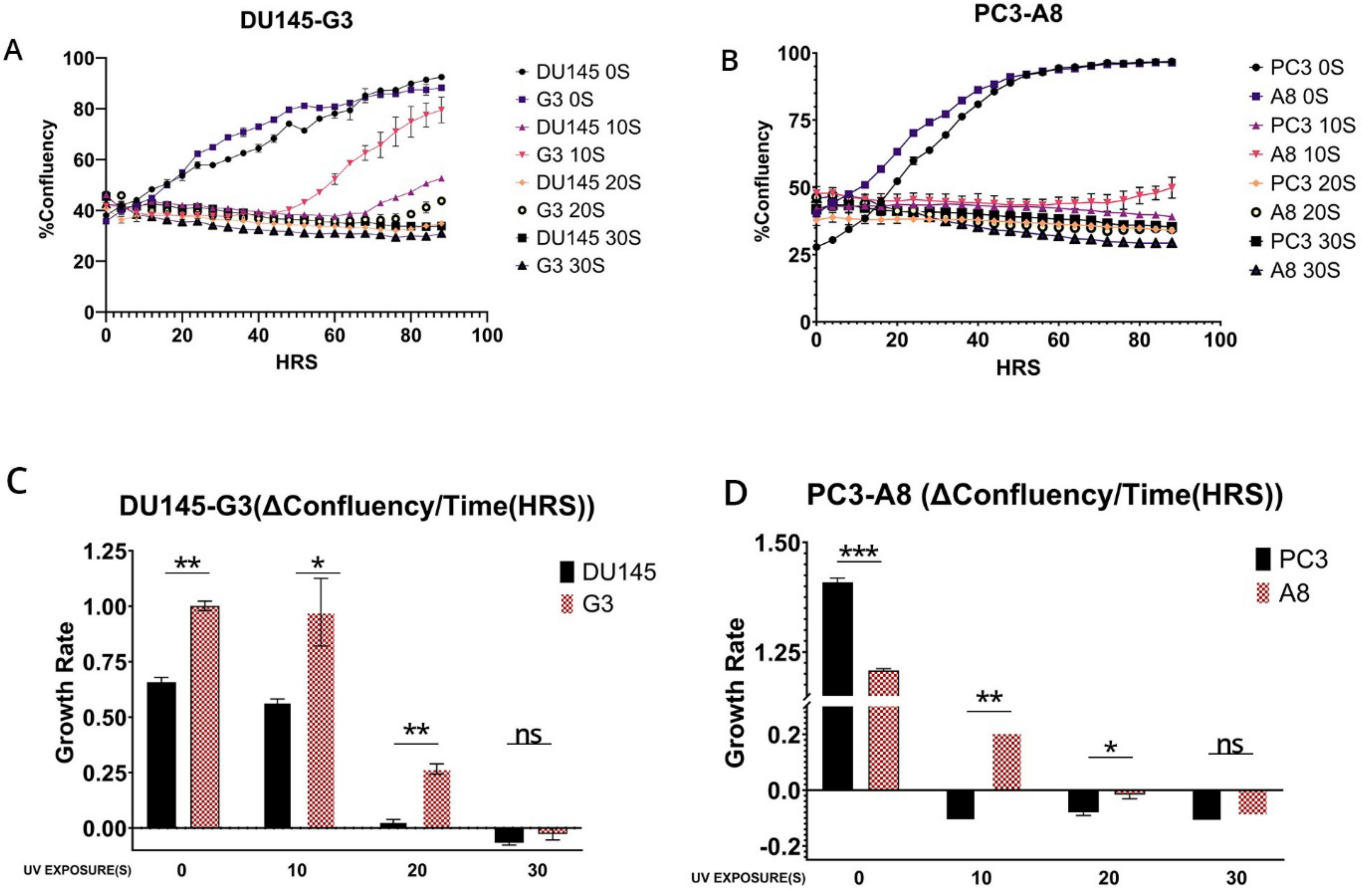
**(A, B)** Proliferation assay of the wild-type and M1097V mutants following exposure to UV at different time points and recovery for 4 days. **(C, D)** Rate of change in cell number – confluency (%) to time (HRS) *p*-value: *p* < 0.05*, *p* < 0.01**, and *p* < 0.001***.

**Figure 4:**
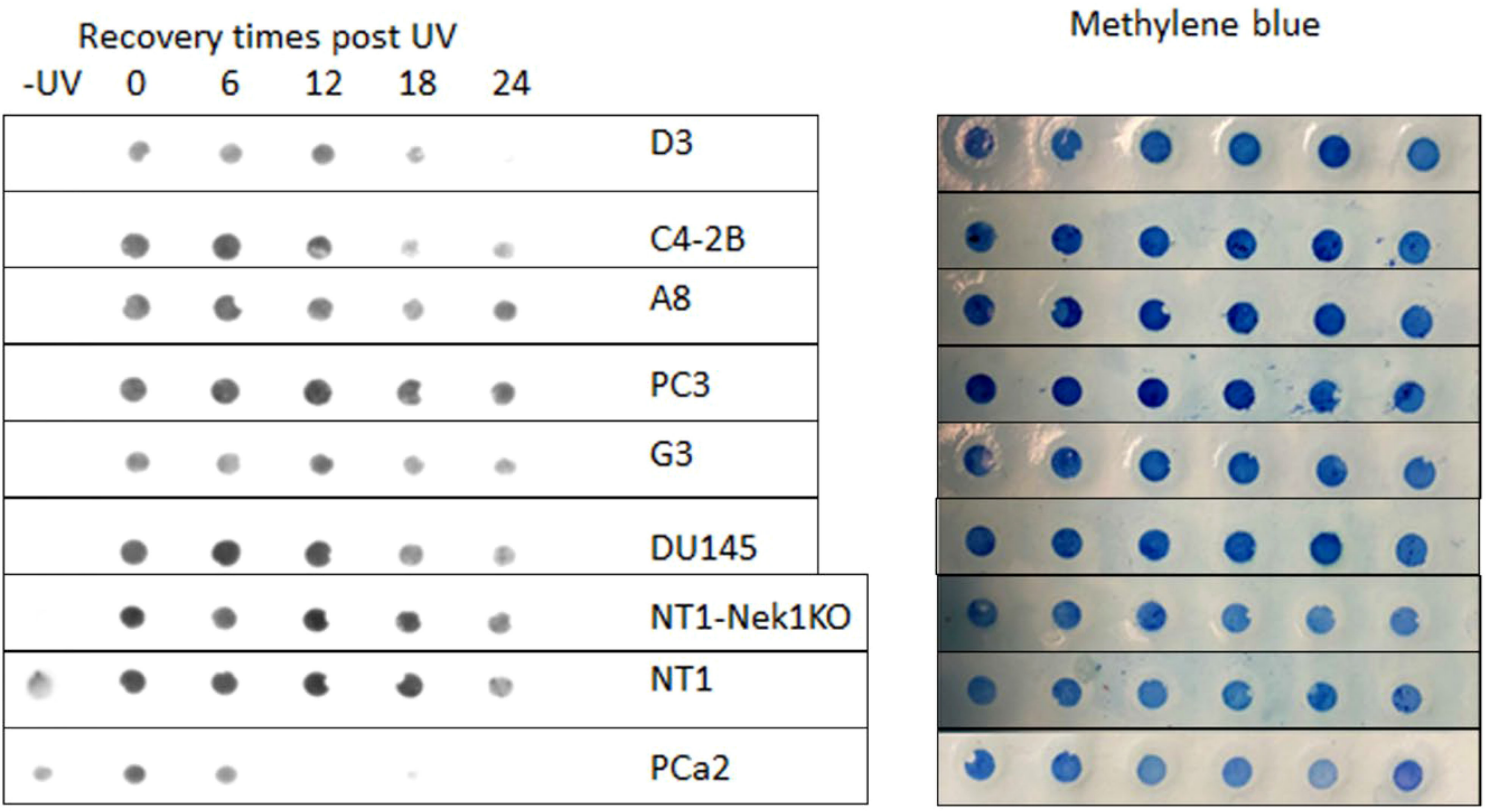
Kinetics of CPD removal. Cells were irradiated with UV (200 mJ/cm^2^) and allowed to recover for the indicated times. DNA was isolated and affixed to Hi-Bond via a manifold. Following brief staining with MB to ensure even DNA application, the blot is probed with CPD antiserum.

**Figure 5:**
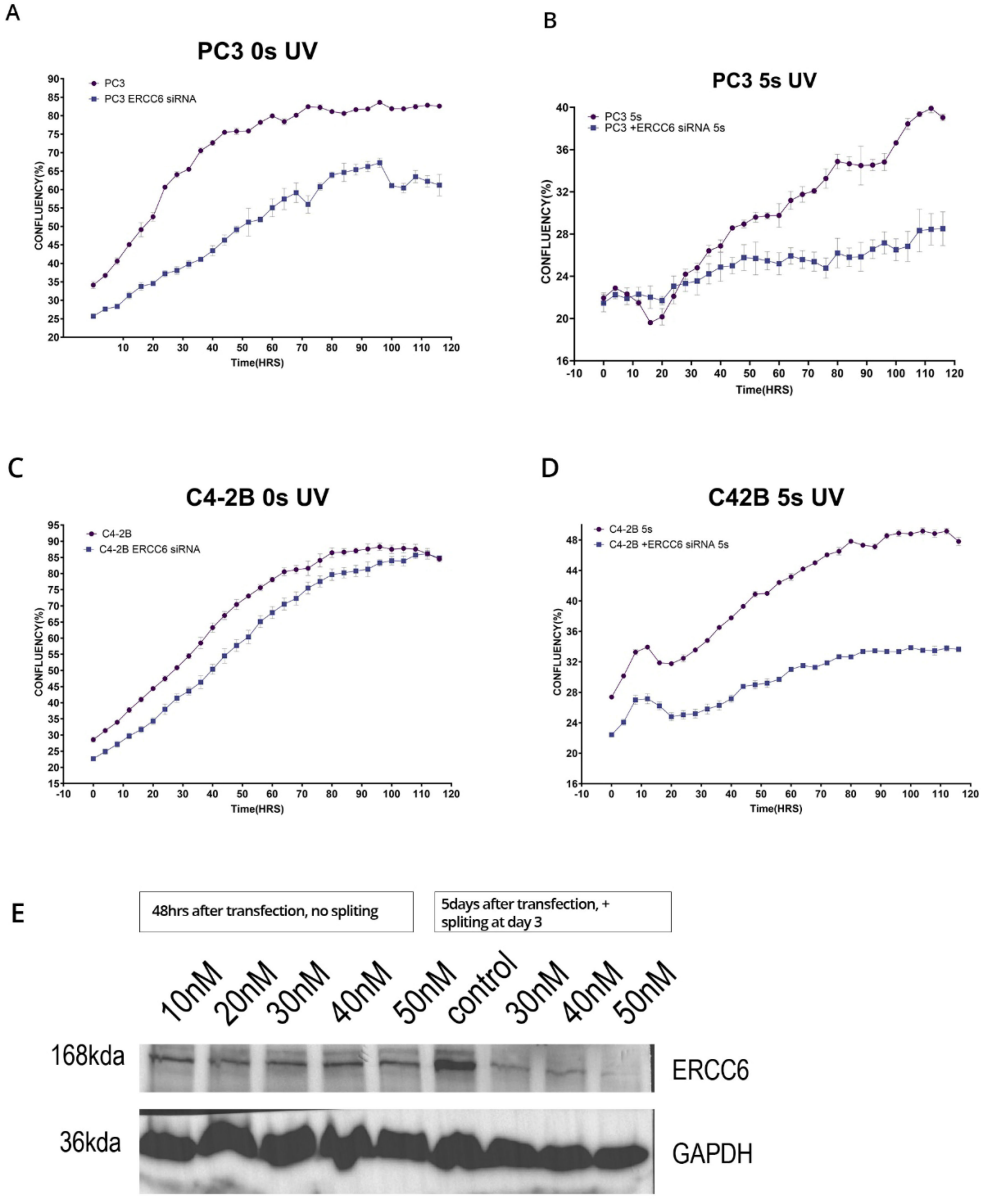
**(A–D)** Proliferation assays in parental cells treated with siRNA and exposed to 5 s UV. **(E)** WB of representative knockdown of ERCC6 (ERCC6/CSB antibody (24291-1-AP)Proteintech) after treatment of cells with siRNA, in this case Hek293, but we obtained similar results with PC3 and DU145. Separately, we noticed that while siRNA treatment results in rapid depletion of ERCC6 mRNA, for the efficient protein turnover, the cells needed to be dividing and not confluent/quiescent, as depleting the critical ERCC6 mRNA expression may result in emergency compensatory protein-stabilization mechanisms in quiescent cells (quantitations in the **Supplementary Materials**).

We assessed the UV and CDDP sensitivity dependence on the ERCC6 mutation in these derivate clones, and surprisingly (against our initial hypothesis), we found that the M1097V mutation conferred somewhat greater resistance to UV doses (**Figure 3**) and faster resolution of UV-induced CPDs (**Figure 4**). Interestingly, the PCa2 line from an AA patient, which surprisingly carries an ERCC6 mutation (Y776C), also showed remarkable activity in CPD removal when compared to all other lines (**Figure 4**). This clearly depends largely on ERCC6, as siRNA-KD for it drastically reduced their survival from UV (**Figure 5**). For control, we also used our NT1-Nek1-KO cells (18), where we know that Nek1 (a key substrate of TLK1) phosphorylates and activates ERCC6 [not published—although ERCC6 was previously reported as a target of Nek4 (19)], and these showed almost no repair (removal of CPDs) even after 1 day. The faster CPD repair activity in the ERCC6-M1097V mutants and PCa2 cells forced us to rethink our initial hypothesis of the greater sensitivity to bulky/distorting lesions damage (mainly CPDs) in AA subpopulations, but only by changing a bit of our point of view. In practice, an “overactive” TC-NER mechanism can be more mutagenic (under the right conditions) than an underactive, deficient one (more in Discussion).

During NER strand replacement/polymerization at the incision site, there is a significant chance of introducing mismatches if the bulky lesions are elevated, particularly in the presence of 8-oxoguanine (8OG). It may not be so surprising that mismatch repair (MMR) can be more mutagenic than NER-mediated correction of bulky lesions due to the lack of precise “mutated strand” discrimination during MMR (20); hence, the ERCC6 variants found in AA-PCa can still be targeted by the NER and HRR combination strategy, as these are expected to yield more DSBs during cisplatin inter-/intrastrand crosslinks (CDDP-ISLs) incision/processing. In short, UV sensitivity (orchestrated via CPD removal) and CDDP sensitivity (via complex combination pathways that include NER and HRR) are not overlapping as one may think, and CSB may be beneficial in one and not the other type of damage.

As there are no available cell lines carrying the M1097V mutation, we have introduced this via CRISPR-mediated editing/recombination. After the generation of a panel of clones for each cell line, we proceeded with their analysis, as shown in the example in **Figure 2**. The introduced mutation removes one of the two Hind3 cleavage sites, so that the WT sequence gets cleaved by Hind3 into two products of the ~500-bp PCR product (lanes 1 and 2, top gel), but if it is mutated, it does not. Note that in the well from clone D3(C42B) and A8(PC3), there is an intact band + the two cleavage products (at ~300 bp and 100 bp PCR product), which indicates a heterozygous SDM change. In contrast, G3 is a genuine (bi-allelic) homozygous SDM. We have begun assessing the UV sensitivity dependence on the ERCC6 mutation in these derived clones, and surprisingly (against our initial hypothesis), we found that the M1097V mutation conferred greater resistance to UV doses (**Figure 3**) and faster resolution of UV-induced CPDs (**Figure 4**). Interestingly, the PCa2 line from an AA patient, which surprisingly carries an ERCC6 mutation (Y776C), showed remarkable activity in CPD removal when compared to all other lines. For control, we used our NT1-Nek1-KO cells (18), where we know that Nek1 (a key substrate of TLK1) phosphorylates and activates ERCC6 [unpublished; although ERCC6 was reported as a target of Nek4 (19)], and these showed almost no repair (removal of CPDs) even after 1 day. The faster NER activity in the ERCC6-M1097V mutants and in PCa2 cells made us rethink our initial hypothesis for the greater sensitivity to bulky lesion (e.g., CPDs) damage in AA subpopulations exposed to the Louisiana toxic, historically racial, disparity environment (15, 16), but only by changing a bit our point of view (see Discussion).

To establish if the rate of clearance of the CPD translates to differences in survival/fitness, we performed a proliferation assay following exposure to UV using the PCa cells and their ERCC6 M1097V counterparts. Without UV exposure, the ERCC6 M1097V mutant achieved confluency faster than the WT variant, and at 10 s UV exposure, there is a clear difference between both PC3 and DU145 and their M1097V counterpart’s survival rates (**Figure 3**). Since UV exposure results in a cell cycle arrest, there is an expected recovery lag before proliferation resumes, but more importantly, when it does, the slope of proliferation rate gives a key estimate of how good the repair was, as an indication of the overall “fitness’’ of the cells’ population after recovery from a highly mutagenic event. When we compare the slopes in the plot, at 10 s UV exposure, G3 is growing at roughly 2× the rate of the WT variant once past the recovery lag, and similarly for A8. In conclusion, the ERCC6-M1097V variant, introduced by SDM replacement at the natural genomic location with its expression regulated at the normal level, confers better TC-NER activity on UV-induced CPDs and greater survival/repair fitness.

Since it is critical to establish that these differences in recovery capacity are attributable directly to ERCC6 repair activity, which inherently differ between the WT and M1097V variant, we performed a viability assay in cells in which ERCC6 was knocked down and exposed to a low dose of UV. This experiment demonstrated that although there are other mechanisms to deal with UV damage (CPDs), including GG-NER, ERCC6-mediated TC-NER is still the most critical repair pathway (**Figure 5**).

**Figure 6:**
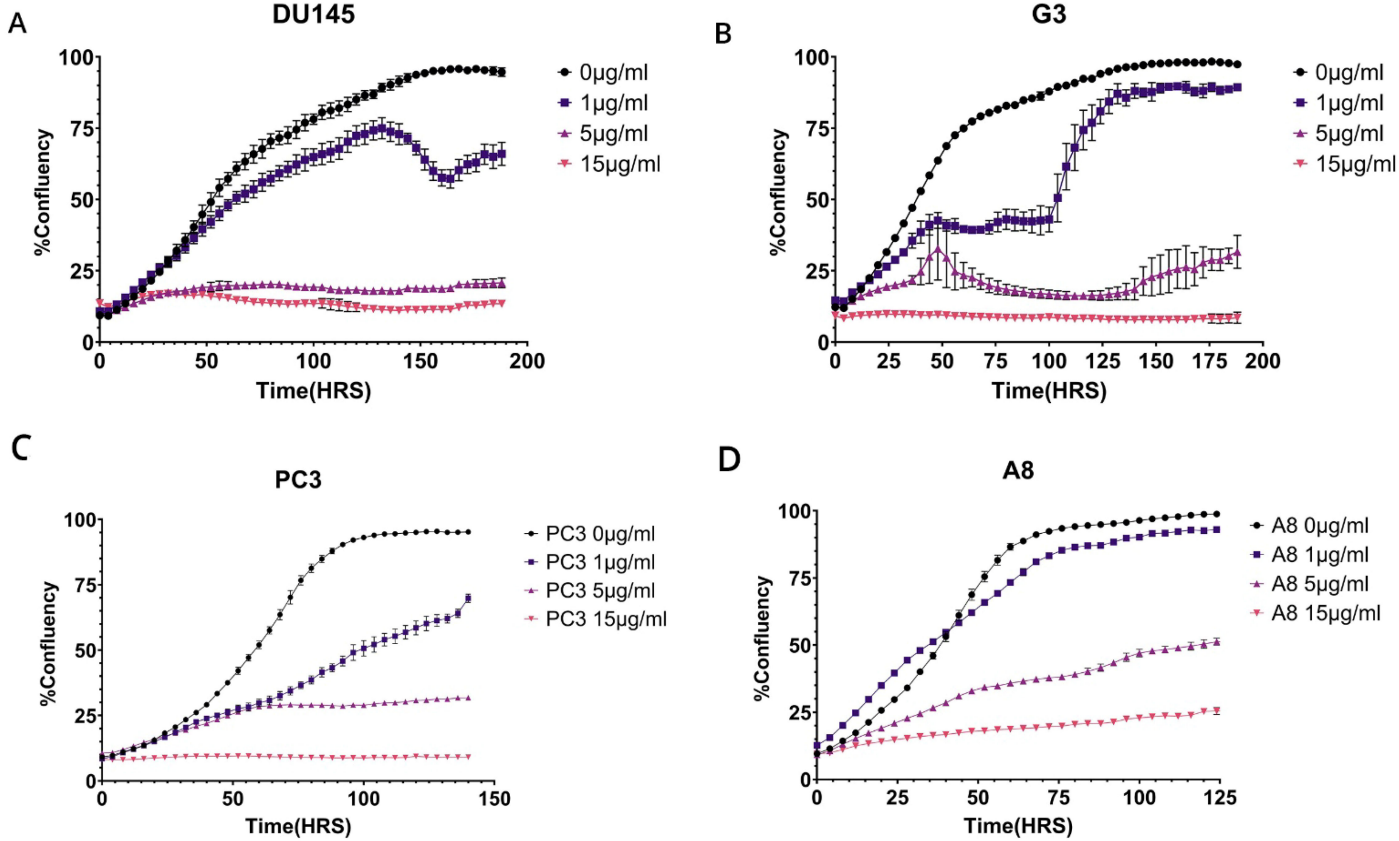
**(A–D)** Proliferation assay of PC3 and DU145 wild-type and M1097V mutants following exposure to different CPT doses. The cells were pretreated with 1, 5, and 15 μg/mL cisplatin (CPT) for 6 h, then washed and replated in 96-well plates for monitoring growth with the IncuCyte over the next several days, as indicated.

We have proposed that the better resolution of CPDs and recovery from UV mutagenic toxicity afforded by the M1097V variant could instead result in worse survival from more complex lesions like those from cisplatin (CDDP, e.g., ISLs) that require the involvement of multiple repair mechanisms. In **Figure 6**, we have set to determine this. We had previously established a range of CDDP concentrations that work best for DU145 and PC3 (21), so we exposed the parental cells and their congenic M1097V mutants to three doses of CDDP for 6 h, and then monitored for viability over the next several days. The result was that the pattern of CDDP sensitivity for the M1097V mutants depends on the cellular context. For the DU145 mutant cells (G3), their recovery (best illustrated at 1 μg/mL CDDP) was severely impaired for several days. However, after day 5, these cells showed almost complete growth-rate recovery capacity, suggesting that the M1097V mutant can eventually elaborate a significantly better TC-NER response, although as explained in the Discussion, this cannot be biochemically studied directly from the endogenous isolated protein. In contrast to the G3, at 1 μg/mL CDDP, the parental DU145 cells were barely affected, although there was an apparent population collapse after 6 days upon reaching 50% well confluence. Since these cells continued unabated growth when untreated, it may suggest some genotoxin-induced trained effect on a contact inhibition checkpoint that normally does not occur in DU145 (they can actually grow on top of each other). At 5 μg/mL CDDP (and above), viability was lost for both parental and mutant no cells.

For PC3 and the M1097V derivative (A8), the situation was different. At all concentrations of CDDP, the A8 mutant cells did considerably better in viability, indicating again that the M1097V mutant can perform better repair of even more complex CDDP-induced lesions.

**Figure 7:**
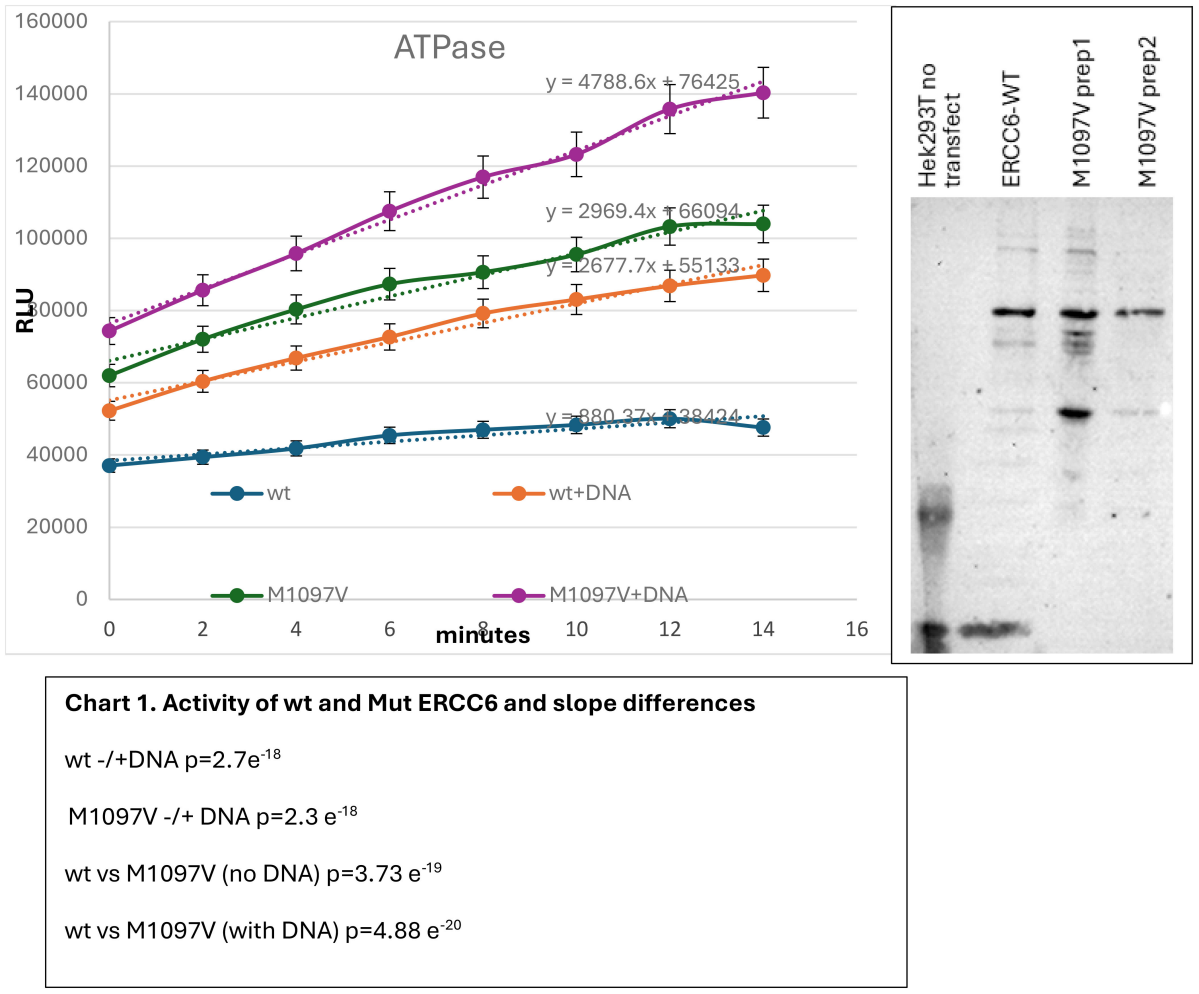
ATPase activity of purified CSB. ERCC6-wt and M1097V mutant were expressed in 2×10^6^ Hek 293T cells by transient transfection and affinity purified with Myc epitope antiserum followed by adsorption on protein A beads. ATPase kinetics (min) were carried out with 200 nM ATP in supplied buffer and ±50 ng plasmid DNA. *Y*-axis: Arbitrary RLUs. ATPase kinetics are followed with ADP-Hunter+ (with supplied stop solution) in triplicates.

To better understand the reason for the better CPD repair activity by the M1097V mutant, we have begun preliminary studies with a high-level mammalian expression vector (Origene-RC219020); after SDM, we IP’d wt and M1097V mutant Myc-tagged-ERCC6 expressed from Hek293 cells, as previously detailed (17), and monitored its DNA-dependent ATPase (22–24). As shown in **Figure 7**, we found that its DNA-stimulated ATPase ratio was twofold greater for the wt, but the basal intrinsic ATPase rate (*K*_cat_: min^−1^) was significantly higher for the M1097V mutant (Chart 1)—the question “how would this different rate/pattern for the variant impact survival from UV *vs*. CDDP?” remains. We should also point out that the plasmid DNA substrate was “undamaged” (not UV irradiated), considering that the ERCC6 association would be unaffected by this aspect, and that there was no stalled Elongation Complex (EC) in these reactions that needed to be displaced as part of the main ATP expenditure in real life.

In the inset, 1/10 affinity-purified proteins are shown on a silver-stained 8% SDS-PAGE.

## Discussion

ERCC6 (also known as CSB protein) is a gene encoding a critical protein involved in DNA repair, specifically in TC-NER. Helix-distorting DNA lesions that are dealt with in this pathway include CPDs, bulky adducts (including cisplatin), and, in some instances, modified or oxidized bases like 5OG. Many more roles have been ascribed to this large protein that belongs to the class of helicases/translocases. In fact, ERCC6/CSB is a multifunctional protein that couples transcription to DNA repair, remodels chromatin, regulates gene expression, and helps maintain genomic and cellular integrity, especially in the face of damage that disrupts transcription of PolI and PolII (https://www.genecards.org/cgi-bin/carddisp.pl?gene=ERCC6).

General activities of ERCC6/CSB:

1. Transcription-coupled DNA repair (TC-NER)

o ERCC6 plays a central role in detecting and initiating the repair of DNA lesions that block transcription.
o When RNA polymerase II stalls at DNA damage (such as UV-induced CPDs), ERCC6 helps recruit repair factors to remove the lesion and resume transcription.

2. Chromatin remodeling

o ERCC6 possesses ATP-dependent chromatin remodeling activity, allowing it to alter nucleosome positioning.
o This activity is crucial for providing repair machinery access to DNA in compact chromatin regions.

3. Transcription regulation

o In addition to DNA repair, ERCC6 can regulate gene expression by interacting with transcription machinery.
o It influences RNA polymerase 1 and RNA polymerase II pausing and restart, ensuring proper transcription resumption after repair.

4. Interaction with other repair proteins

o ERCC6 interacts with other TC-NER factors such as CSA (ERCC8), XPG, TFIIH, and UVSSA to coordinate the repair process.

5. Response to oxidative stress

o ERCC6 has been implicated in the repair of oxidative DNA damage, not just UV-induced lesions.
o It helps maintain mitochondrial function and cellular redox balance under stress conditions.

6. Role in disease

o Mutations in ERCC6 cause Cockayne Syndrome, a rare autosomal recessive disorder characterized by growth failure, neurodegeneration, and premature aging.
o It is also linked to other neurodevelopmental and progeroid syndromes.

While loss-of-function (LOF) mutations result in severe syndromes, the function of most missense mutations (or variants) has not been well studied, nor are they understood. The M1097V polymorphism was identified as a significant risk for ontogeny and worse outcome in meta-analyses of several types of cancer, particularly kidney, worldwide (12–14), and more recently noted as a significant incidence recurrence in a study of possible racial disequilibrium in Louisiana AA patients with PCa. However, the functional significance of this ERCC6 variant and its activity in PCa development or progression was unknown. The fact that there are very few established PCa cell lines from AA that can be used as representative of their typical repairome forced to approach this problem by introducing this mutation, at the correct genomic location and functional regulation, in a panel of the most common PCa cell lines, including PCa2 derived from an AA patient, and that interestingly already carries a different mutation (Y776C) in ERCC6 of unknown significance. We should add that in our limited exome study of a patient with PCa from the more northern region of Louisiana, we did not identify the M1097V mutation, although our collection included an S636N missense variant that resides very closely to the SYSY-624-628 “hydroxy patch” that we have found to be highly phosphorylated by Nek1 and strongly regulates the activity of ERCC6 after UV damage (17). The close proximity of the S636N on the same loop fold suggests that the variant may affect the activity of the protein, although it is hard to predict in which direction, but we did study the activity of the M1097V variant, and perhaps unexpectedly, it increased the tolerance to UV of all the cell lines in which we carried out the appropriate gene replacement. The potential implications for this are significant, considering some segments of the Louisiana population have remained local for generations, with potential implication for high sunlight exposure and a regional toxic environment of Louisiana, dubbed “cancer alley” (25). In this respect, it is important to emphasize that an advantageous mutation against UV utilizing TC-NER does not necessarily protect against more complex lesions partially utilizing this repair pathway, like those caused by petrochemically derived aromatic alkylating agents or, in the case we have studied, CDDP that also causes ICLs that require the action of several pathways in addition to NER for completion of repair. In fact, the opposite could happen from an overzealous TC-NER that can lead to the accumulation of mismatch mutations during replacement of the damaged “flap” of DNA. In practice, an “overactive” TC-NER mechanism can be more mutagenic (under the right conditions) than an underactive/deficient one. During NER strand replacement/polymerization at the incision site, there is a significant chance of introducing mismatches when bulky lesions are numerous, particularly in the presence of 8OG. It should not be so surprising that MMR can be more mutagenic than NER-mediated correction of bulky lesions due to the lack of precise “mutated strand” discrimination during MMR (20). In this regard, we should note that it was remarkable how many LOF mutations were found among a panel of MMR genes in our repairome study of patients with PCa (unpublished), in both AA and CC, clearly supporting the concept of the “mutator phenotype” in cancer (26) championed by Louis A. Loeb. Hence, the ERCC6 variants found in AA-PCa can still be successfully targeted by an NER and HRR combination strategy (e.g., with a synthetic lethal combination of CDDP and J54), as these are expected to yield more DSBs during CDDP-ISLs processing.

AAs are at higher risk for developing PCa than CC and have a more aggressive disease that is refractory to treatment (3), to some extent explained by genetic differences and in some cases attributed to alterations of the “repairome” (4). Since AR signaling regulates the growth, survival, and proliferation of prostate tumors, the majority of PCa therapies are focused on either inhibition of androgen synthesis or blockade of AR transactivation. However, the drug effect does not last long and the tumor relapses within 18–24 months with a more aggressive phenotype known as mCRPC. Androgen ablation, radiotherapy, and chemotherapy are commonly employed for the treatment of both localized PCa and mCRPC. Inflicting DNA damage and enhancing apoptosis of cancer cells are the mechanistic strategies of all radiotherapeutic (e.g., external beam radiation therapy, brachytherapy, and radium-223) and some chemotherapeutic (PARP inhibitors; topoisomerase inhibitors, platinum-based therapy (5), and DNA crosslinking agents) methods (6, 7). The relative success of PARPi for patients with mCRPC with and without homologous recombination repair (HRR) mutations (PROfound (8) and TRITON2 (9)) is defining new biological and more effective treatment options for previously unmanageable PCa, and while cisplatin (CDDP)-based therapy is currently not the treatment of choice for PCa, partly due to its significant renal toxicity [which can be faithfully recapitulated in mice (10)], the current trend suggests that lower dosing in combination with inhibitors of DNA damage repair could be quite effective (5). In Louisiana, PCa disparity is far more prevalent, with higher incidence and worse OS that can be attributed to both genetic components and dietary habits (15), compounded by a greater health risk from a regional toxic environment that was built to disproportionally impact the AA population (16). We see many of these patients presenting to LSU Health Shreveport with advanced stage disease both in the north and from a central region sadly named “cancer alley” (25). Perhaps paradoxically, the more frequent germline and somatic mutations in NER genes identified in AA (11) that may be negatively impacted from this toxic environment and that are likely germane to higher mutation-induced cancer are also key to a therapeutic strategy that could be largely beneficial to AA patients with PCa. Alterations of key genes involved in NER could result in incomplete or aberrant repair of the bulky lesions (or ISLs) and result in potentially lethal effects for the cancer cells. This relies on a synthetic lethality approach simultaneously targeting the TC-NER and HRR pathways. In particular, we propose that one could target a function TLK1 as a regulator of RAD54 activity and the availability of some novel specific inhibitors like J54 (27–29) as a potential synthetic lethal approach we have recently employed (21). In contrast, in this work, we have focused our efforts to direct a future synthetic lethal therapy designed for the more active TC-NER activity of one specific haplotype variant of the gene ERCC6 that is frequently found in mCRPC of AA patients (11), reportedly a germline. Such combination therapy approaches may bypass altogether the need for androgen deprivation therapy (ADT) (i.e., castration) and its significant side effects that few men would readily choose, or clearly for those who would not respond to ADT/ARSI from the start, as epitomized by NEPC cases, which is also a more common occurrence for AA patients with PCa, in addition to the already mentioned higher incidence of mutations in their repairome (30). Of note, since NEPC is refractory to AST/ARSI from the onset, these patients are eligible for CDDP as standard of care early on (31). We did not test for differences in the pattern of resistance to common environmental base alkylating agents like benzopyrene, or oxidative damage, which can be dealt with by multiple pathways and mostly via base-damage-specific BER (like for 8OG). However, we did carry out viability studies after cisplatin as the most relevant TC-NER lesion type for this paper, with our “genomic replacement cells”. We decided that CDDP (causes ICLs as well as single-base adducts) should be sufficient for this initial study, as we hypothesized it could offer a possible alternative treatment option for patients with PCa carrying this variant. The results from the CDDP tolerance were dependent on cell context. This should not be surprising as CDDP causes a number of different lesions [intrastrand crosslinks, interstrand crosslinks (ICLs), monofunctional adducts, DNA–protein crosslinks]. In fact, although CDDP has not yet entered the standard of care for PCa, its success has been reported in patients with advanced PCa when studied in the context of DNA repair gene aberrations (5). If we accept the M1097V variant as a “better protein” for performing TC-NER, it will still have to be part of the subsequent interaction with the other repair machineries, which are specific to each PCa cell, that ultimately translate in their survivability even though ERCC6 was reported to be the principal player in repair of CDDP lesions based on a CRISPR-KO screen (32). The significant lag in eventual growth recovery demonstrated in the G3 cells warrants further studies, as it could be a potential Achilles heel for patients with PCa carrying this mutation when designing a cogent multi-drug therapy exploiting this liabilities, as it was also reported that adding CDDP can restore sensitivity to ARSI of some CRPC cell lines or improve response to taxanes (33, 34).

For supporting direct biochemical studies, we did not address this, but the endogenous expression of ERCC6 is quite low and further complicated by the existence of a unique and notable example of a transposon fusion involving the ERCC6 gene with the formation of the ERCC6-PGBD3 gene that is instead highly expressed (SI-WB). A PiggyBac transposable element derived 3 (PGBD3) integrated into an intron (intron 5) of the ERCC6 gene. This occurred approximately 43 million years ago before humans and marmosets diverged. As a result of this insertion, the ERCC6 gene now produces not only the normal full-length ERCC6 protein but also a fusion protein called ERCC6-PGBD3. This happens through alternative splicing, where the first 5 exons of ERCC6 are spliced to the entire PGBD3 sequence. It is thus nearly impossible to obtain reliable biochemical assays, like DNA-dependent ATPase from IPs of cells expressing these “genomic variants”. We have thus begun studies with a high-level mammalian expression vector (Origene-RC219020) and we hope to have more solid biochemical assays, such as DNA translocase activity and cellular phenotypic information (DDRR), from these variants in the future. Our initial DNA-dependent ATPase comparisons are intriguing, but similar detailed studies with Mfd (the *E. coli* ERCC6 paralog) revealed that really complex ATPase cycles (some DNA-independent) promote large infrastructural rearrangements while displacing the “stuck EC” (35) (see Video S1). Also, in future studies, we will include the identification of PDX tumors with ERCC6 variants from our patients’ population and test them *in vivo* for sensitivity to cisplatin treatment. In addition, although our current IRB approval is limited to studies of de-identified subjects, subsequent plans are to include PHI in follow-up studies.

## Conclusions

Our findings underscore the critical and multifaceted role of ERCC6/CSB in DNA repair and transcription regulation, particularly through the TC-NER pathway. The M1097V variant of ERCC6 enhances UV resistance in PCa cells, suggesting a possible evolutionary adaptation with modern therapeutic implications. This variant, along with other ERCC6 alterations found predominantly in AA patients with PCa, could be exploited using synthetic lethality strategies targeting TC-NER and HRR. Such precision therapies may be especially beneficial in regions like Louisiana, where environmental exposures and inherited variants converge to heighten cancer risk and severity.

## Data Availability

The raw data supporting the conclusions of this article will be made available by the authors, without undue reservation.
