# Supplementary figures and images for: Studies on the functionality of the TC-NER ERCC6-M1097V protein variant frequently found in Louisiana patients with PCa upon UV damage
Source: Front Oncol. 2026 Feb 3;15:1679379. doi: 10.3389/fonc.2025.1679379 (PMC12909189; doi:10.3389/fonc.2025.1679379)

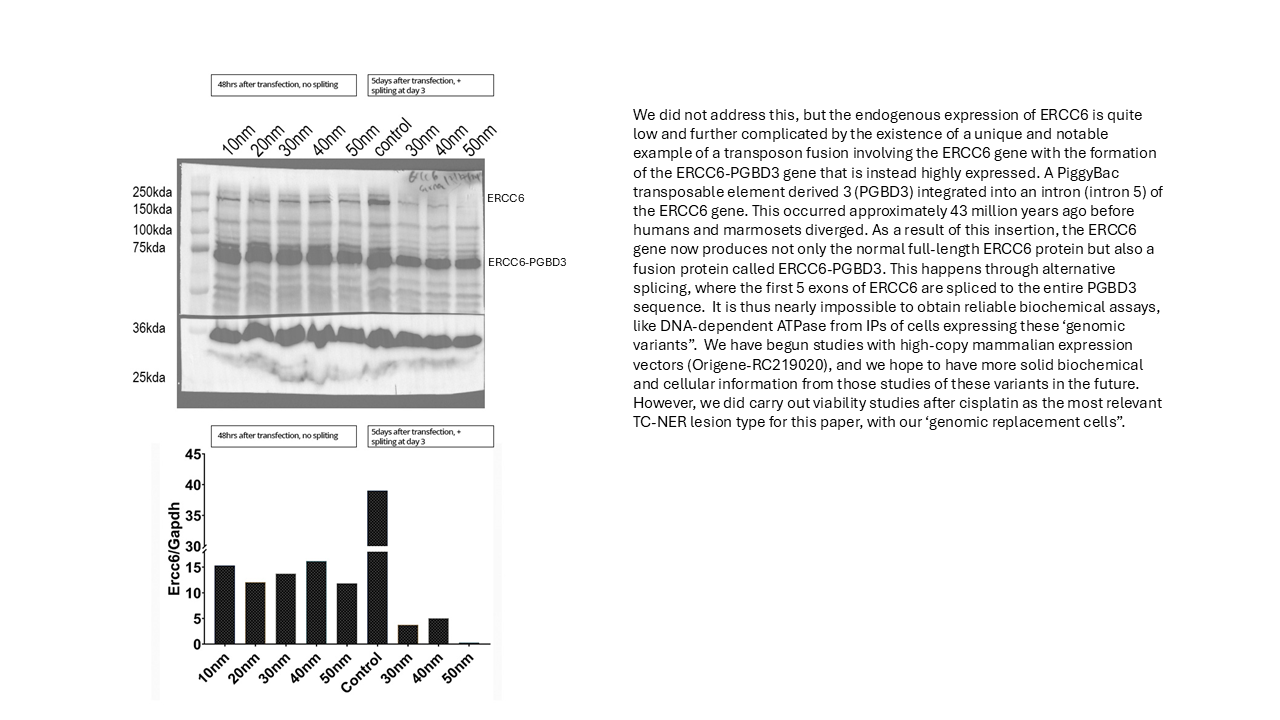

Supplement: Supplementary file 1 [file Image1.tif]

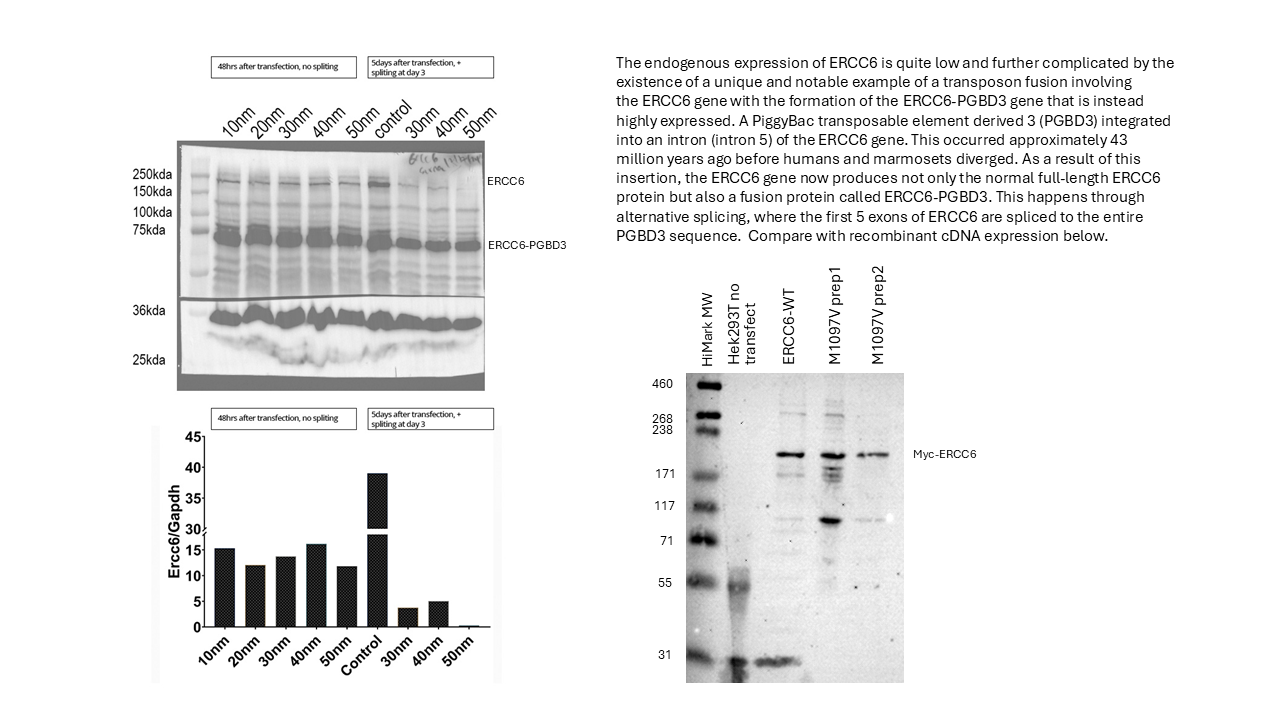

Supplement: Supplementary file 2 [file Image2.tif]
